# Chronic Cardiac Herniation: A Peculiar Diagnosis

**DOI:** 10.7759/cureus.56339

**Published:** 2024-03-17

**Authors:** Juan D Ayala Torres, Santiago Andrés Gómez Salazar, Juan Gonzalo Vélez Zuluaga

**Affiliations:** 1 Radiology, Universidad de Antioquia, Medellín, COL; 2 Radiology, Pablo Tobón Uribe, Medellín, COL

**Keywords:** pericardium, magnetic resonance imaging, tomography, diagnostic imaging, pericardiectomy, cardiac herniation

## Abstract

The presented case describes a 56-year-old male with adult-onset Still's disease, exhibiting polyserositis in 2019, who underwent pleurectomy and pericardiectomy. Despite treatment with tocilizumab and methylprednisolone, the patient developed deep vein thrombosis and pulmonary embolism in 2022, managed with apixaban. A contrast-enhanced chest tomography revealed no recurrent thromboembolic events. Over a year, the patient experienced progressive dyspnea, correlating with signs of constriction on transthoracic echocardiogram. Cardiac magnetic resonance imaging confirmed cardiac herniation, prompting pericardiectomy. Surgery led to complete resolution of anatomical alterations without heart failure or new abnormalities, although exertional dyspnea persists post-discharge. The pathophysiology of cardiac herniation involves complex mechanisms influenced by congenital or acquired factors, resulting in abnormal heart protrusion. Medical literature highlights varied presentations, with acute cases typically post-thoracic surgeries, while late-onset cases are less common. Imaging modalities like computed tomography (CT) and cardiac magnetic resonance (CMR) aid diagnosis, emphasizing interdisciplinary collaboration. Despite challenges posed by its rarity, timely diagnosis and treatment are crucial for favourable outcomes, demonstrating the importance of considering this entity in clinical practice.

## Introduction

The cardiac herniation through the pericardium is a rare but potentially serious condition [1]. It is associated with congenital defects and, to a greater extent, trauma and transpericardial procedures [2]. This condition, sparsely documented in medical literature, presents a wide variability of clinical manifestations primarily depending on the size and location of the defect [2-4]. It is usually observed within the first 24 hours following a procedure, with few cases exceeding this time threshold. It is a potentially life-threatening condition that requires timely intervention to ensure the survival of the affected individual [1-5]. Here, we present a particular case of a patient with cardiac herniation that persists for a markedly longer period than described in the literature, associated with a latent symptomatology whose approach through clinical and imaging studies allowed for an appropriate outcome.

## Case presentation

**Figure 1:**
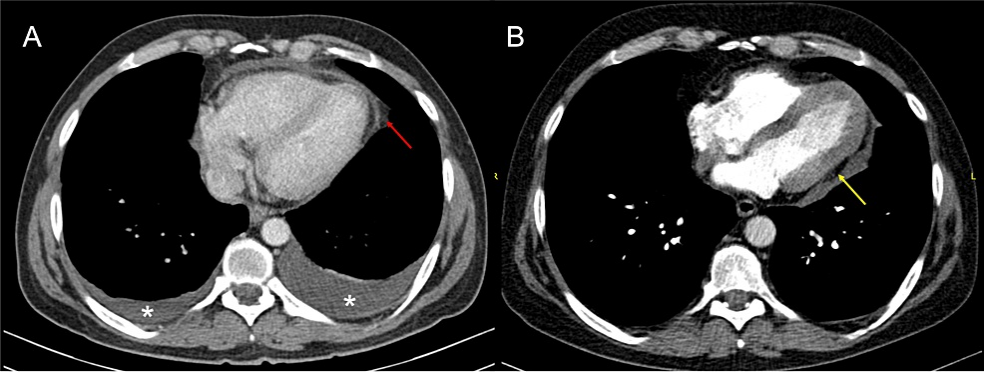
Contrast-enhanced chest tomography A. Contrast-enhanced chest tomography from 2019: Pre-biopsy study of the pericardium. Pericardial thickening and enhancement (Red arrow); Bilateral pleural effusions (asterisk). B. Contrast-enhanced chest tomography from 2022: Abnormal heart shape, with loss of the "cone" configuration of the left ventricle (Yellow arrow).

**Figure 2:**
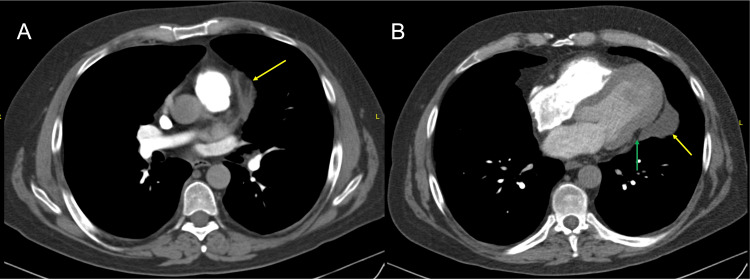
Contrast-enhanced chest tomography Axial slices at the level of the major vessels (A) and at the cardiac base (B), showing thickening of the fibrous pericardium with abnormal thick tissue at the surgical resection margin (Yellow arrows). Abnormal heart shape, loss of the "cone" configuration of the left ventricle (Green arrow), increased compared to previous study.

A 56-year-old male patient with a diagnosis of adult-onset Still's disease, which manifested with polyserositis in 2019 (Figure 1A) and underwent pleurectomy and pericardiectomy. The patient was being treated with tocilizumab and methylprednisolone. In 2022, the patient developed deep vein thrombosis and pulmonary embolism associated with antiphospholipid syndrome, treated with indefinite anticoagulation with apixaban, on that occasion, a new contrast-enhanced chest tomography was performed for diagnostic purposes (Figure 1B), with no recurrence of thromboembolic events to date. The patient has been experiencing dyspnea on exertion for one year, which has progressed to minimal exertion one month prior to the consultation. During the hospital stay, pulmonary embolism was ruled out (Figure 2), and a transthoracic echocardiogram (TTE) was requested, revealing preserved left ventricular ejection fraction (LVEF) with signs of constriction (Video 1), possibly related to cardiac herniation.

**Video 1:** Transthoracic echocardiography, four-chamber apical view It shows abnormal movement of the left ventricle, with extrinsic compression between the basal and mid segments of the lateral wall.

**Figure 3:**
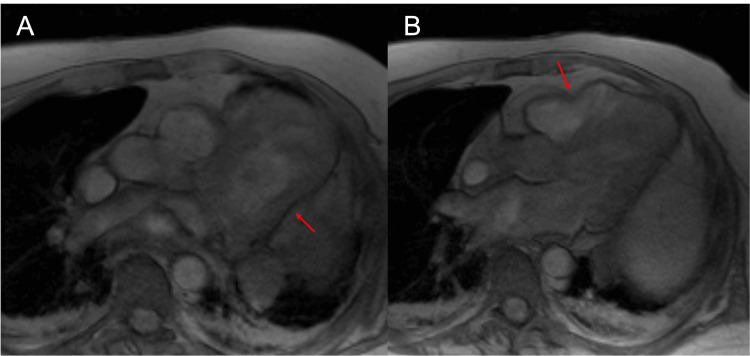
Cardiac magnetic resonance imaging, axial gradient echo (GRE) sequence Pericardial defect with herniation of both ventricles. Notch in the contour of the ventricular margins at the level of the pericardial defect (Red arrows). There are no calcifications.

**Figure 4:**
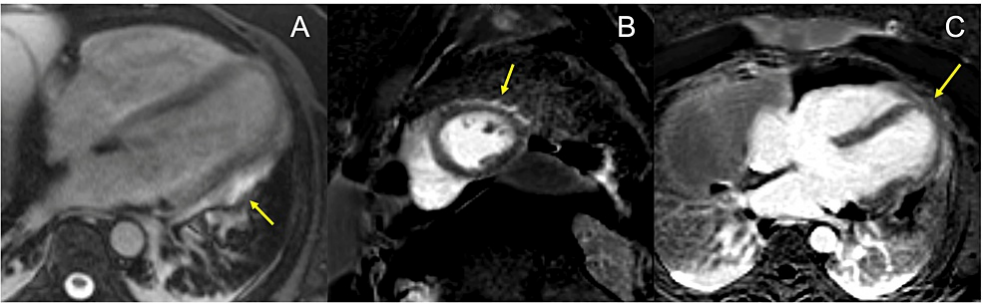
Cardiac MRI A. Cardiac MRI axial post-contrast True Fast Imaging with Steady-State Free Precession sequence: Thickening and enhancement of the pericardial edge (Yellow arrow) coinciding with morphological alteration site in the lateral wall of the left ventricle (Red arrow in A). B and C. Cardiac MRI Phase-Sensitive Inversion Recovery late enhancement sequence: No significant fibrosis at herniation site (Yellow arrow).

It was compared with previous images from 2021 (echocardiogram) that already showed similar findings, so a cardiac magnetic resonance imaging was requested in order to properly characterize the pericardial alteration, confirming the suspicion of herniation (Figures 3, 4), leading to evaluation by the cardiovascular surgery team, who decided to proceed with pericardiectomy for the patient.

**Figure 5:**
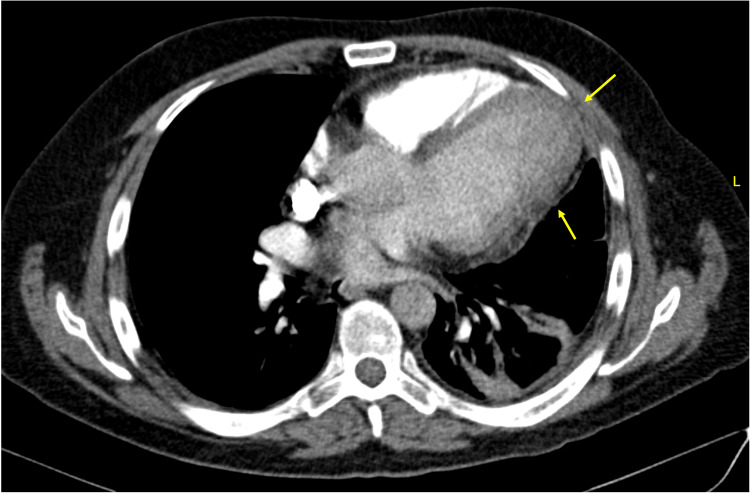
Contrast-enhanced chest tomography Post-pericardiectomy. Improvement of the morphological alteration of the left ventricle (Yellow arrow).

The patient underwent left anterior thoracotomy, with release of multiple adhesions and resection of a broad segment of pericardium above the left and right phrenic nerves, completely releasing the heart. The patient's clinical course was favorable, with no clinical symptoms of heart failure or new abnormalities on echocardiogram and follow-up computed tomography (Figure 5), demonstrating complete resolution of the anatomical alteration that prompted the studies and diagnosis of cardiac herniation. Upon discharge, the patient still experiences exertional dyspnea, so outpatient medical management and cardiac rehabilitation are offered.

## Discussion

The pathophysiology of cardiac herniation involves a series of complex mechanisms leading to the abnormal protrusion of the heart through the pericardial sac. This condition can be congenital or acquired, with development influenced by genetic, environmental, and traumatic factors [1,2,6]. In congenital cases, anomalies during embryonic pericardial development may predispose to cardiac herniation formation, while acquired hernias typically arise from trauma or previous thoracic surgeries [1,2,6]. Regardless of etiology, the underlying pathophysiological process involves an imbalance in the forces acting on the heart and neighboring structures. Herniations can occur in both small and large defects, usually following thoracic surgeries on the same side of the defect where pleural pressure is reduced, causing the heart to protrude through the pericardium [1,2]. Other risk factors for herniation include areas with increased heart mobility and limited supporting tissue around it [2]. Once herniation occurs, the heart may become trapped in an abnormal position, compromising coronary and systemic blood flow, as well as cardiac function [3,4]. Compression of neighboring structures, such as the superior vena cava or pulmonary trunk, can result in symptoms of right or left heart failure, depending on the location and extent of the hernia, leading to serious complications such as vascular obstruction, cardiac ischemia, arrhythmias, and, in extreme cases, death [2,3].

Medical literature describes a variety of cases of cardiac herniation, ranging from asymptomatic presentations to potentially life-threatening complications [3,7]. The most common presentation corresponds to acute cases following thoracic surgeries where pericardial continuity has been breached, whose abrupt presentation, clinical context, and imaging support guide diagnosis and timely treatment [3,4]. On the other hand, more insidious presentations of late onset are unusual and sparsely reported [3-5,7]. Our case presents an unusual presentation due to its prolonged duration and the scarce presentation of symptoms months prior to its surgical correction. Some late cardiac herniations up to six months post-surgery have been reported [3-5,7]. Symptoms typically develop abruptly due to ventricular or great vessel extrinsic compression in the majority of scenarios [3,4,7]. However, similar to our patient, Holloway et al. reported a case with progressive dyspnea over a period of two months following heart and lung transplantation [5]. There are some theories explaining its late appearance, among which the partial adhesion of the pericardium to the heart walls after some incision stands out, which over time tends to give way and facilitate cardiac herniation [3,4]. The disruption of the pericardial sac, as mentioned previously, is the primary trigger of this pathology, with most cases associated with therapeutic interventions [3-5,7], but to date none with diagnostic intention (pericardial biopsy) as in our case.

Radiological findings of cardiac herniation may vary depending on the imaging modality used and the site and extent of heart protrusion. Abnormal mediastinal displacements, such as a poorly defined or displaced cardiac silhouette, can be observed on chest X-ray, as well as indirect signs such as elevation of the pulmonary hilum or heart rotation [2,8]. Computed tomography (CT) and cardiac magnetic resonance imaging (CMR) offer detailed visualization of cardiovascular anatomy, including precise localization and extension of the hernia, as well as identification of functional alterations and possible complications such as compression of neighboring structures [2,3]. The imaging findings, combined with interdisciplinary planning and organization during the patient's latest admission, facilitated a favorable outcome. However, it is worth noting that many of the imaging findings were already present in previous studies, highlighting the importance of strict patient follow-up to detect subtle changes that may guide diagnosis.

## Conclusions

Cardiac herniation through the pericardium poses significant clinical challenges due to its rarity. Each case highlights the complexity and the need for careful medical attention. The present case represents a late presentation that, although very uncommon, underscores the importance of considering this entity. Success in diagnosis and treatment is facilitated by collaboration among clinicians, radiologists, and surgeons.
